# Neural timing of stimulus events with microsecond precision

**DOI:** 10.1371/journal.pbio.2006422

**Published:** 2018-10-26

**Authors:** Jinhong Luo, Silvio Macias, Torbjørn V. Ness, Gaute T. Einevoll, Kechen Zhang, Cynthia F. Moss

**Affiliations:** 1 Department of Psychological and Brain Sciences, Johns Hopkins University, Baltimore, Maryland, United States of America; 2 Faculty of Science and Technology, Norwegian University of Life Sciences, Ås, Norway; 3 Department of Physics, University of Oslo, Oslo, Norway; 4 Department of Biomedical Engineering, Johns Hopkins School of Medicine, Baltimore, Maryland, United States of America; University of Salamanca, Spain

## Abstract

Temporal analysis of sound is fundamental to auditory processing throughout the animal kingdom. Echolocating bats are powerful models for investigating the underlying mechanisms of auditory temporal processing, as they show microsecond precision in discriminating the timing of acoustic events. However, the neural basis for microsecond auditory discrimination in bats has eluded researchers for decades. Combining extracellular recordings in the midbrain inferior colliculus (IC) and mathematical modeling, we show that microsecond precision in registering stimulus events emerges from synchronous neural firing, revealed through low-latency variability of stimulus-evoked extracellular field potentials (EFPs, 200–600 Hz). The temporal precision of the EFP increases with the number of neurons firing in synchrony. Moreover, there is a functional relationship between the temporal precision of the EFP and the spectrotemporal features of the echolocation calls. In addition, EFP can measure the time difference of simulated echolocation call–echo pairs with microsecond precision. We propose that synchronous firing of populations of neurons operates in diverse species to support temporal analysis for auditory localization and complex sound processing.

## Author summary

We routinely rely on a stopwatch to precisely measure the time it takes for an athlete to reach the finish line. Without the assistance of such a timing device, our measurement of elapsed time becomes imprecise. By contrast, some animals, such as echolocating bats, naturally perform timing tasks with remarkable precision. Behavioral research has shown that echolocating bats can estimate the elapsed time between sonar cries and echo returns with a precision in the range of microseconds. However, the neural basis for such microsecond precision has remained a puzzle to scientists. Combining extracellular recordings in the bat’s inferior colliculus (IC)—a midbrain nucleus of the auditory pathway—and mathematical modeling, we show that microsecond precision in registering stimulus events emerges from synchronous neural firing. Our recordings revealed a low-latency variability of stimulus-evoked extracellular field potentials (EFPs), which, according to our mathematical modeling, was determined by the number of firing neurons and their synchrony. Moreover, the acoustic features of echolocation calls, such as signal duration and bandwidth, which the bat dynamically modulates during prey capture, also modulate the precision of EFPs. These findings have broad implications for understanding temporal analysis of acoustic signals in a wide range of auditory behaviors across the animal kingdom.

## Introduction

Diverse groups of animals, such as electric fish, owls, and echolocating bats show remarkable temporal precision in the processing of sensory events. Specifically, weakly electric fish can detect a stimulus time disparity on the order of 0.4–1 μs [1,2], barn owls can distinguish an interaural time difference of approximately 10 μs, and big brown bats (*Eptesicus fuscus*) can discriminate differences in echo arrival time on the order of 36–80 μs in target range discrimination tasks [3–5] and <1 μs (and down to 10 ns) in target range jitter discrimination tasks [6–8]. The neuronal basis of the microsecond temporal precision has been identified for both electric fish and barn owls [9–12]. Specifically, single neurons in the prepacemaker nucleus of the weakly electric fish were found to be sensitive to temporal disparity as small as 1 μs [11]. Similarly, the firing rate of many neurons in the midbrain of the barn owls can distinguish an interaural time difference smaller than the behavioral threshold [9]. By contrast, the neural basis for microsecond auditory resolution in echolocating bats remains unknown.

Neurons hypothesized to function in bat sonar target distance measurement show facilitated responses to pairs of sounds, separated by a restricted range of delays, which mimic bat sonar calls and echoes, and this response property is referred to as echo delay tuning [13–15]. Although it has been hypothesized that echo delay–tuned neurons encode target range information in bats [16–19], the tuning widths of echo delay–tuned neurons in echolocating bats are typically several milliseconds wide [13,14,17,20], and this is far beyond the behavioral threshold.

It has also been hypothesized that the variability in response latency of auditory neurons may contribute to the bat’s sonar range resolution [21–25]. In contrast to echo delay–tuned neurons with millisecond delay tuning widths, the response latency of many auditory neurons of echolocating bats varies by only a few hundred microseconds, a reduction in timing errors of up to a factor of 100 [24]. For example, about one-third of neurons in the midbrain inferior colliculus (IC) of the big brown bat show latency variability <1 ms [26]. The most precise neurons in the bat IC show latency variability between approximately 100 and 250 μs [23,25,26]. Of note, neurons of submillisecond latency precision are not exclusive to echolocating bats but have also been reported in other animal models, such as cats and mice [27,28].

How echolocating bats discriminate echo arrival time with microsecond resolution remains an unsolved problem [18,19,29–31]. Here, combining extracellular recordings and mathematical modeling, we show that synchronous neural firing can improve the precision of stimulus event timing an order of magnitude greater than the temporal precision of single neurons. Of note, in this study, we analyzed the stimulus-evoked extracellular field potential (EFP, 20–600 Hz) to characterize neural timing in a band up to 600 Hz, whereas local field potential (LFP) is generally analyzed in a lower band, up to 100–200 Hz. The most precise EFPs (in the 200–600-Hz band) showed a latency variability of 17 μs, based on extracellular recordings in the IC of seven awake big brown bats passively listening to simulated echolocation calls. Moreover, the spectrotemporal structure of the echolocation calls affected the latency variability of the EFP, which is consistent with predictions from sonar receiver models of ranging accuracy [32–34].

## Results

We conducted three experiments with a total of seven bats. The first experiment investigated response latency variability measurements with a standard two-harmonic frequency modulated (FM) stimulus of 3-ms duration and approximately 80-kHz bandwidth, the second experiment investigated the influence of stimulus time–frequency structure on response latency variability, and the third experiment investigated the response latency variability measurements with simulated call–echo pairs.

### Reliable detection of EFPs with microsecond precision

**Fig 1:**
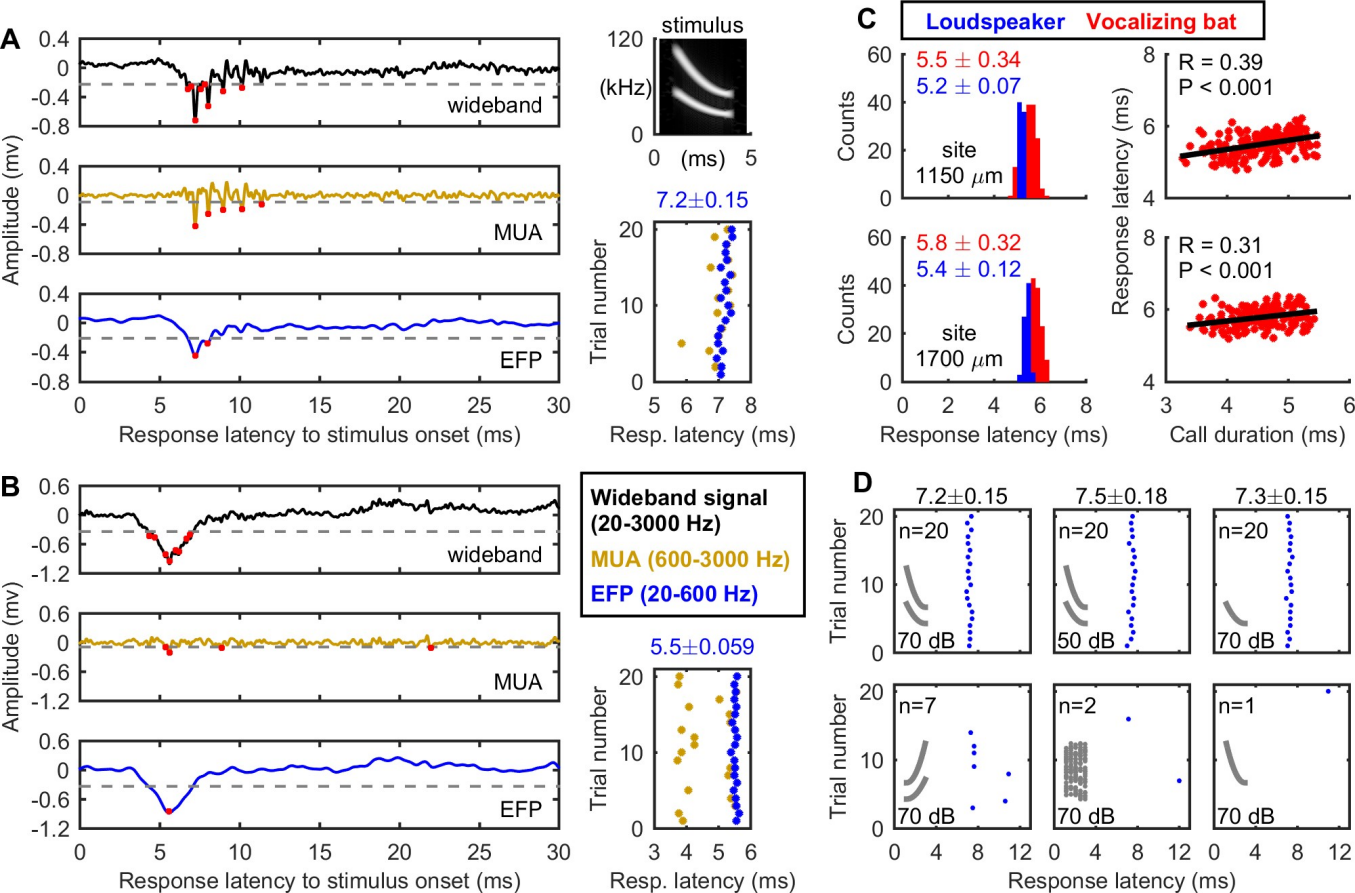
Echolocation calls evoke precise EFP in bat IC. (A, B) Two example neural recordings from a bat (R63) illustrating peaks (red dots) identified from the wideband signal (black), MUA (orange), and EFP (blue) by an adaptive threshold (gray dashed line). Both EFP and MUA were derived from the same wideband neural recording with different cutoff frequencies for the digital filters. The right column shows the spectrogram of the stimulus, the “standard” call (top), and the response latencies of the first peaks. The “standard” call was 3 ms in duration, 70-dB SPL in amplitude, and consisted of two quadratic harmonics with the first harmonic sweeping down from 55 kHz to 25 kHz. (C) Similar response latencies of EFPs were observed when the bat (R14) was either listening to simulated echolocation calls from a loudspeaker or spontaneously vocalizing bats. (D) Response latencies of EFPs were selective for stimulus parameters. Number *n* indicates the total number of EFP peaks detected over 20 trials, i.e., 20 repetitions of the same acoustic stimulus (gray inset). All stimuli were 3 ms in duration. The first harmonic swept down from 55 kHz to 25 kHz and the second harmonic from 110 kHz to 50 kHz. The white noise was from 110 kHz to 25 kHz. Response latency is not shown for the lower panels of (D) due to the very few responses. Data shown here were from a single bat (R63) and data from all three bats were shown in S1 Fig and S1 Data. All response latency data are presented as mean ± SD. EFP, extracellular field potential; IC, inferior colliculus; MUA, multiunit activity; SPL, sound pressure level.

In the first experiment, we took multichannel extracellular recordings with silicon probes in the IC of three head-fixed, awake big brown bats (one male, two female) passively listening to the broadcasts of simulated wideband echolocation calls of 3-ms duration and 70-dB sound pressure level (SPL) amplitude (Fig 1A, top right) (referred to as the “standard call” in this study). Based on a threshold for detection of six times the background noise floor, corresponding to a signal-to-noise ratio (SNR) of 15 dB (Fig 1A, grey dashed line), we recorded stimulus-evoked EFPs (20–600 Hz) of large amplitude (Fig 1A, blue trace) in addition to the spikes from multiunit activity (MUA) (Fig 1A, orange trace). Note that both EFP and MUA were derived from the same wideband neural recording with different cutoff frequencies of the digital filters. Over 20 presentations of the standard 3-ms FM stimulus, the response latency of the first EFP, measured as the time difference between the stimulus onset and the first negative peak of the EFP, was very stable, with a standard deviation (SD) of 150 μs in this example. Fig 1B shows data from another recording site that did not pick up isolated spikes but instead high SNR EFPs, with a latency SD of 59 μs. From both examples and S1 Movie, one can see that EFPs occurred directly after the presentation of the echolocation calls and typically occurred once per sound presentation. The high-amplitude, temporally precise EFPs have not been reported in neurophysiological studies of auditory processing in echolocating bats.

To address the possibility that the EFPs were artifacts of electrical noise from our sound broadcast system, we made neural recordings from the IC of a bat listening to two spontaneously vocalizing conspecifics inside the sound booth. Further, this approach allowed us to study responses to variable, natural stimuli, which animals process in the real world. Fig 1C shows that EFPs were reliably evoked in response to the natural vocalizations of bats in the recording booth. Compared to the broadcasts of acoustic stimuli through an electrostatic loudspeaker, the natural bat vocalizations resulted in slightly longer response latencies and greater variability, which can be explained by differences in call parameters, particularly the amplitude and duration differences between sonar playbacks and natural sounds. In the loudspeaker broadcasts, the stimulus amplitude (70-dB SPL) and duration (3 ms) were fixed. By contrast, the calls produced by the spontaneously vocalizing bats varied in duration by approximately 2.2 ms (3.3–5.5 ms) and amplitude by approximately 30 dB. The fact that EFPs could be evoked by vocalizations of live bats when the electrostatic loudspeaker was not powered eliminates the possibility that EFPs recorded in this study were a result of electrical artifacts. Moreover, EFPs showed selectivity for the fine spectrotemporal structure of the acoustic stimuli. Fig 1D shows EFPs from one recording site: EFPs were reliably detected in response to the top-row stimuli, including the standard call, −20 dB of the standard call, and the first harmonic of the standard call, but not in response to the bottom-row stimuli, which included a time-reversed version (upward FM sweep) of the standard call, white noise, and the second harmonic of the standard call. S1 Fig shows stimulus selectivity by the EFP for data from all three bats (S1 Data). There were 202 recording sites, in which at least one of the six types of the acoustic stimuli evoked ≥5 EFP responses over 20 presentations (i.e., ≥25% response probability). We found that the EFP showed a selective response at 193 (95.5%) and 124 (61.4%) recording sites, based on a 25% and 50% response probability difference between at least two acoustic stimuli, respectively. Thus, the stimulus-evoked EFPs reflect robust responses to biologically relevant acoustic stimuli.

### EFP response latencies are more precise than MUA and single neurons

How are the precise EFPs generated? Synaptic inputs and spiking activity are two main sources of EFPs [35–38]. The contribution of synaptic inputs to EFPs declines rapidly above approximately 200 Hz. Spiking activity, on the other hand, contributes to both the 20–200-Hz band and the 200–600-Hz band [39,40]. Thus, the 20–200-Hz EFPs can arise from both synaptic inputs and spiking activity, while the 200–600-Hz EFPs reflect primarily spiking activity. To assess whether the observed EFPs arose specifically from spiking activity, we separated the 20–600-Hz band EFP into 20–200-Hz band and 200–600-Hz band and analyzed them separately. In total, the 200–600-Hz EFP was detected at 528 recording sites from three bats (154 sites from Bat 1, 126 sites from Bat 2, and 232 sites from Bat 3). By contrast, the 20–200-Hz EFP and 20–600-Hz EFP were detected at 162 and 243 recording sites in total, respectively. The fact that the 200–600-Hz EFP was more prevalent in our recordings suggests that spiking activity contributed to the stimulus-evoked EFP, and all following EFP analyses were carried out on the 200–600-Hz EFP with 90% detection reliability. A 90% detection reliability criterion indicates that EFPs or spikes were detected in at least 72 out of 80 stimulus presentations.

**Fig 2:**
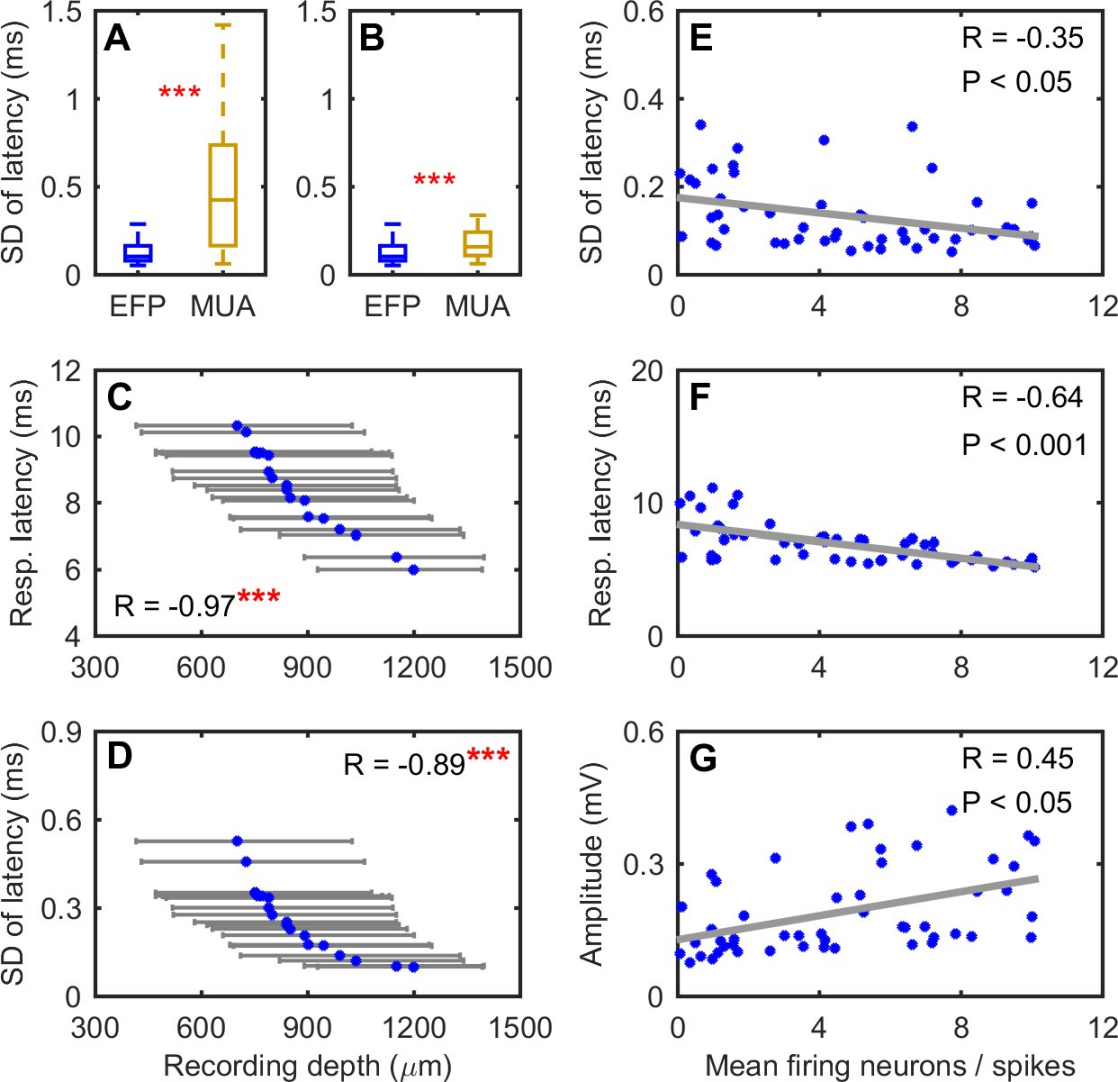
Properties of EFP. (A, B) EFPs (200–600 Hz) are more precise in timing stimulus onset than MUA. Data are based on a 90% detection reliability. The detection reliability refers to the probability at which MUA/EFP peaks were detected across all trials for the same sound stimulus. The MUA data in B were a subgroup of the original data in which values larger than the maximum of the data in the EFP group were excluded. (C, D) Topographical organization of the response latencies of the EFP (200–600 Hz). The recording depth is displayed as the median and quartiles for each detection reliability between 5% and 95% at a step of 5%. As indicated by the asterisks, the median recording depth was significantly correlated with the response latency and its variability. (E–G) The number of firing neurons affects the properties of the EFPs. The number of firing neurons refers to the average number of negative peaks whose amplitudes exceeded the detection threshold of the wideband signal (20–3,000 Hz; see Fig 1A and 1B) within a time window of ±3 ms relative to the averaged latency of the EFP over 80 repetitions. Data for this figure are included in S1 Data. EFP, extracellular field potential; MUA, multiunit activity.

In addition to the EFP, we analyzed the first negative peak of multiunit activity (MUA; 600–3,000 Hz). A comparison of the latency variability between the first negative peak of the EFP and MUA revealed that the EFP latency was more stable than the MUA (Fig 2A; Wilcoxon rank sum test, *P* < 0.001). At the 90% detection reliability, the median SD for the EFP and the MUA were 104 μs (minimum SD: 53 μs) and 425 μs (minimum SD: 62 μs), respectively. A detailed examination of the data sets revealed that the MUA data contained many recording sites with greater latency variability than the EFP data, which might be the basis of the observed population differences. To test this idea, we excluded the sites of the MUA group whose SD were larger than the maximum SD of the EFP data but again found that the EFP response latencies were more stable than the MUA (Fig 2B; Wilcoxon rank sum test, *P* < 0.001). These results show that EFP latency is more precise in timing stimulus events than MUA.

There is a topographical organization of the EFP across the IC with respect to the stability of response latency (Fig 2C and 2D). Specifically, high-precision EFP latency was most often recorded in the ventral region of the IC, whereas higher variability in the EFP latency tended to appear in the dorsal region of the IC (Fig 2D). Similarly, EFPs of shorter response latency were mainly found in the ventral region of the IC (Fig 2C), which aligns closely with earlier observations of single neurons in the IC [41].

The EFP to the 3-ms standard FM stimulus showed a latency variability as low as 53 μs, which is more precise than the least variable single unit latencies of IC neurons reported in the literature, between 100 and 250 μs [23,25,26]. To test whether our recordings contained single neurons that were far more precise than those previously reported, we identified single spiking units of high SNR using the Wave_clus algorithms [42] and carefully evaluated the waveforms of the sorted single units (see S1 Text for details). In total, we identified 59 single units in our recordings. The most precise single unit in our data set had an SD response latency of 140 μs (with a population median of 1.36 ms), which is within the range of values reported in the literature.

### Population size and neural synchrony influence the properties of the EFP

Our analyses revealed that the EFP showed the highest temporal precision in response latency, and single units showed the highest latency variability. This raises a fundamental question: how does the activity of single neurons contribute to EFPs of greater temporal precision? Since action potential waveforms only contain a proportion of energy in the EFP frequency band (<600 Hz), multiple overlapping spikes are required to produce a prominent EFP. We hypothesized that the number of firing neurons and neural synchrony influence the temporal precision of the EFP.

To gain insights into the effect of the number of firing neurons on the properties of the EFP, we counted the number of negative peaks in the wideband neural signal, whose amplitudes were larger than the detection threshold (20–3,000 Hz, Fig 1A and 1B), within ±3 ms of the mean response latency of the EFP. We chose the ±3-ms time window to count the number of firing neurons, as it largely overlaps with the 5-ms period of the 200-Hz high-pass cutoff frequency of the EFP, aiming to obtain counts of neurons potentially contributing to the EFP. We counted the number of firing neurons from the wideband neural signal (20–3,000 Hz) rather than from the 600–3,000-Hz band (i.e., spikes), since spikes from neurons at a distance that cannot be picked up by the electrode also contribute to the low frequencies of the EFP [35,36,39,43]. In other words, the 20–3,000-Hz wideband neural signal may contain more information than the MUA. We found that there was a weak negative correlation between the number of firing neurons and the temporal variability of the EFP (Fig 2E). Moreover, the number of firing neurons correlated negatively with the response latency and positively with the peak amplitude of the EFP (Fig 2F and 2G). These results suggest that the number of firing neurons could influence the properties of the EFP. The weak correlations between the number of firing neurons and the properties of the EFP also imply that the number of firing neurons is not the only factor influencing the properties of the EFP.

### Simulations and mathematical models explain the properties of the EFP

**Fig 3:**
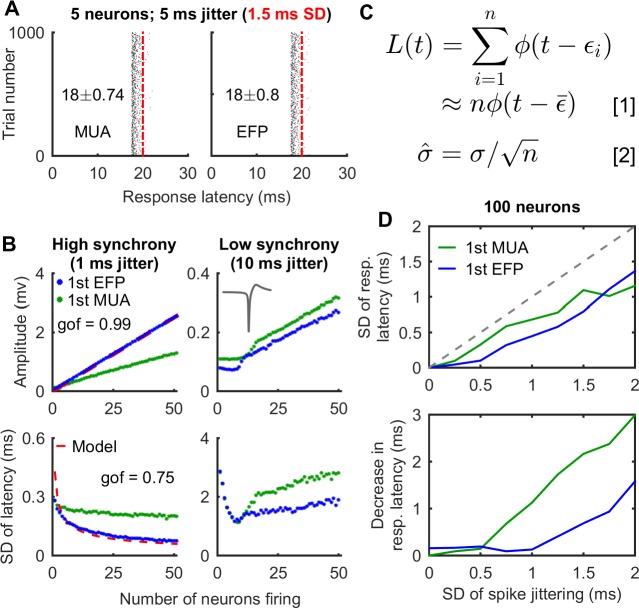
Mathematical models may explain the properties of EFP. (A) In computer simulations, both MUA and EFP showed shorter average response latency and more precise timing than single neurons. The average response latency of single neurons (20 ms, red dash-dot line) itself does not affect the temporal precision of either the MUA or EFP and was chosen arbitrarily for illustrative purposes. (B) In computer simulations, MUAs and EFPs became larger in amplitude and more precise in timing with an increasing number of neurons firing synchronously. With an increasing number of neurons, EFPs showed better temporal precision than MUA for the high-synchrony scenario (left column). Red dashed line shows the predicted effect of the number of firing neurons on the EFP through least squares fitting of the simulation data based on the mathematical models presented in figure panel C (see S1 Text for details). The gof represents the adjusted r-square of least squares fitting. (C) Equation 1 shows that the amplitude of the EFP (*L*(*t*)) increases linearly with the number of firing neurons (*n*). Equation 2 shows that the SD of EFP latency ($\hat{\sigma }$) is proportional to the SD of single neurons (*σ*) and decreases with increasing number of neurons (*n*), following a square root law. (D) Biophysical simulations with 100 neurons firing at varying degrees of synchrony, based on a cell model from Neocortical Microcircuitry [44]. The first peaks of both MUA and EFP had better temporal precision and shorter response latency than the single neurons. Importantly, the EFP was better in temporal precision than the MUA when neurons fired in high synchrony. EFP, extracellular field potential; gof, goodness of fit; MUA, multiunit activity.

To systematically investigate the effect of the number of neurons and neuronal firing synchrony on the EFP properties, we took a simulation approach that allowed us to examine quantitatively the effect of one factor at a time. We started with the simplest scenario in which five neurons with the same extracellular spike shape fired an action potential at a random latency between 17.5 and 22.5 ms, and thus each neuron had a response latency of 20 ± 1.5 ms (mean ± SD, indicated by the red dash-dot line in Fig 3A). Of note, the 20-ms average response latency is arbitrarily chosen for illustrative purposes and does not affect the simulation results. Then, we band-pass filtered this virtual recording to generate simulated MUA and EFP and analyzed the first negative peak of the MUA and EFP for each simulated trial in the same way as for the experimental data. Fig 3A shows the results of 1,000 simulations. Both the MUA and EFP showed responses that were shorter in response latency and more precise in timing than the single neuron, supporting the experimental observations. Subsequently, we quantified the effect of the number of neurons on MUA and EFP for both high-synchrony scenarios in which all neurons fire randomly within a 1-ms time window and low-synchrony scenarios in which all neurons fire randomly within a 10-ms time window. Fig 3B shows that the MUA and EFP become more precise and greater in amplitude with increasing number of firing neurons but only under the high-synchrony simulation. For instance, the first EFP showed a precision of 75 μs and an amplitude of 2.5 mV when 50 neurons fired within a 1-ms time window (Fig 3B), which is approximately four times more precise and approximately 32 times greater in amplitude than the single neuron. Moreover, the effect of increasing the number of firing neurons is stronger for the EFP than for MUA. Thus, this result is consistent with the experimental observation that the EFP can be more precise than MUA (Fig 2A and 2B).

Moreover, one can mathematically demonstrate that when multiple neurons fire within a short time window, about the width of the action potentials, EFPs become shorter in response latency, more precise in timing, and larger in amplitude (Fig 3C, see details in S1 Text). This mathematical analysis leads to three predictions: (1) the peak amplitude of the EFP increases linearly with increasing number of the firing neurons; (2) the variability of EFP latency decreases as the number of firing neurons increases, following a power law relationship; (3) the decrease in response latency of the EFP is positively related to its variability. Importantly, all of these predictions agree with the experimental observations (Fig 2E–2G) and simulations (Fig 3A and 3B).

The simplified simulations and the mathematical model based on multiple copies of a single extracellular spike shape allowed us to directly examine the influence of the number of firing neurons and neuronal synchrony on the properties of the EFP, yet extracellular recordings from neurons naturally vary in spike waveform, which additionally depends on the position and geometry of the electrode. To consider a more realistic scenario, we performed biophysical simulations in which neurons produced varying spike waveforms at a virtual recording electrode. Fig 3D shows that the conclusions drawn from the simplified simulation approach also hold for simulated recordings with different extracellular potential waveforms. For example, the precision of the EFP latency measurement was higher than that of MUA, and the single neurons showed the highest latency variability. Particularly, the EFP (200–600 Hz) measurement showed a precision of 45 μs with 100 neurons each firing with a 250-μs SD. Moreover, the magnitude of response latency reduction of both MUA and EFP latency measurements correlated positively with the variability of single neurons.

### Functional correlates between EFP and bat sonar behavior

**Fig 4:**
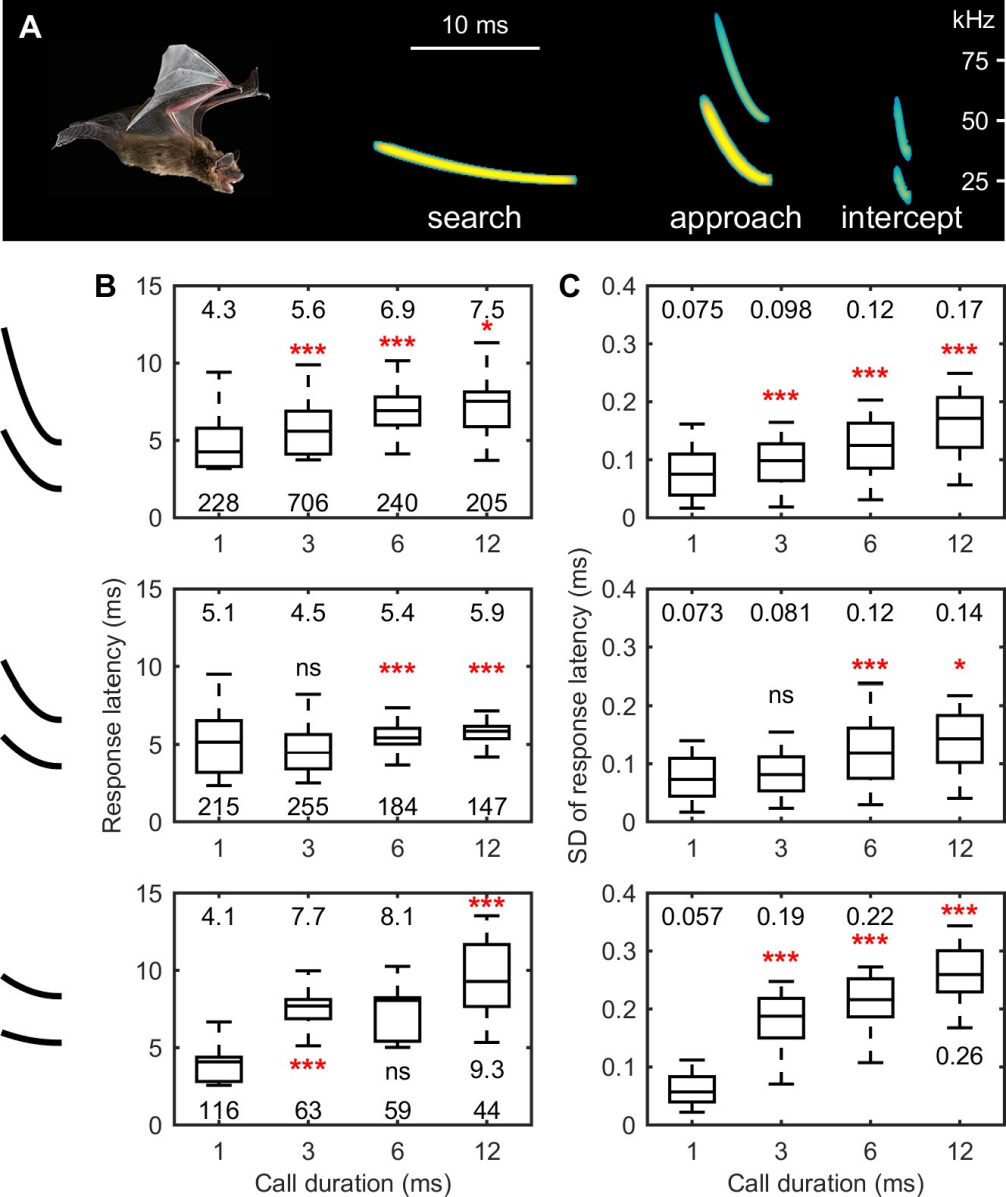
Functional correlates between EFP and bat sonar behavior. (A) Big brown bat dynamically adjusts the spectral–temporal structure of echolocation calls as it searches, approaches, and intercepts the prey. Echolocation calls were replotted from Moss and Surlykke (2001) [47]. (B, C) Echolocation calls of short duration and wide frequency range evoke EFPs of the highest temporal precision and shortest response latency. For each condition, the data were from the EFP sites whose temporal precision was among the top 50% and whose peaks were reliably detected for at least 90% of the trials. The number above each boxplot is the median, and the number below the boxplot in figure panel B is the number of recording sites (i.e., sample size). Statistical significance levels of the nonparametric rank sum test between the data group and the neighboring left data group are indicated by asterisks (*P* < 0.05*, *P* < 0.01**, *P* < 0.001***) or “ns” (*P* > 0.05). Of note, the same effects of call features on EFP properties can be seen in S2 Fig in which the EFPs from the same recording site were compared across signal durations. Data for this figure are included in S1 Data. *The bat image in (A) is credited to B*. *Fenton and B*. *Falk*. EFP, extracellular field potential.

One key component of bat echolocation is the dynamic adjustment in the spectrotemporal features of sonar signals with target distance [45–48]. For example, big brown bats initially use long duration calls (up to 15 ms) of narrow frequency bandwidth (fundamental sweeps over 3–5 kHz) to search for insects in open space and then progressively shorten the duration (down to 1–2 ms) and widen the bandwidth of the calls (fundamental sweeps over 30 kHz) while approaching the prey (Fig 4A). It has been shown that bats achieve the highest precision in sonar ranging with short, wideband calls, which they produce when approaching prey [8,32,49]. Thus, we hypothesized that the precision of stimulus registration with the EFP changes with signal duration and signal bandwidth. Specifically, we predicted that wideband, short echolocation calls (i.e., produced by bats in the prey approach phase) should generate EFPs with the highest temporal precision, and narrowband, long echolocation calls (i.e., produced by bats in the prey search phase) should generate EFPs of the lowest precision.

To test the hypothesis that stimulus bandwidth and duration influence the temporal stability of the EFP latency, we conducted a second experiment by taking extracellular recordings from the IC of four awake big brown bats (all females) passively listening to simulated echolocation calls of varying bandwidth and durations. We found that measurements of EFPs at a single site showed the highest precision in registering the timing of acoustic events (a minimum SD of 17 μs at a recording depth of 1,060 μm) when the bats were listening to echolocation calls of the shortest duration (1 ms) (Fig 4C, S2 Fig). The average value of the 10 most precise EFPs evoked by the 1-ms broadband echolocation calls was 21 ± 2 μs. By contrast, EFPs showed the largest variability in registering the timing of acoustic events (a minimum SD of 168 μs at a recording depth of 720 μm) when the bats were listening to calls of longest duration (12 ms) and narrowest bandwidth (5-kHz bandwidth of the first harmonic). The average value of the 10 most precise EFPs evoked by the 12-ms narrowband echolocation calls was 200 ± 20 μs. Overall, the precision of EFP timing with respect to stimulus events became progressively poorer with increasing call duration (two-way ANOVA, *F* = 158.85, *df* = 3, *P* < 0.001, all *P*_*adj*_ < 0.001 for pair-wise comparisons). Similarly, the response latency of EFPs became progressively longer with increasing call duration (Fig 4B) (two-way ANOVA, *F* = 218.9, *df* = 3, *P* < 0.001, all *P*_*adj*_ < 0.001 for pair-wise comparisons). EFPs to narrowband calls showed the greatest variability in the temporal registration of stimulus events (two-way ANOVA, *F* = 65.63, *df* = 2, *P* < 0.001, all *P*_*adj*_ < 0.001 for pair-wise comparisons) and the longest response latency (two-way ANOVA, *F* = 85.21, *df* = 2, *P* < 0.001, all *P*_*adj*_ < 0.001 for pair-wise comparisons), except for the shortest duration of 1 ms (Wilcoxon rank sum test, all *P*_*adj*_ > 0.05).

**Fig 5:**
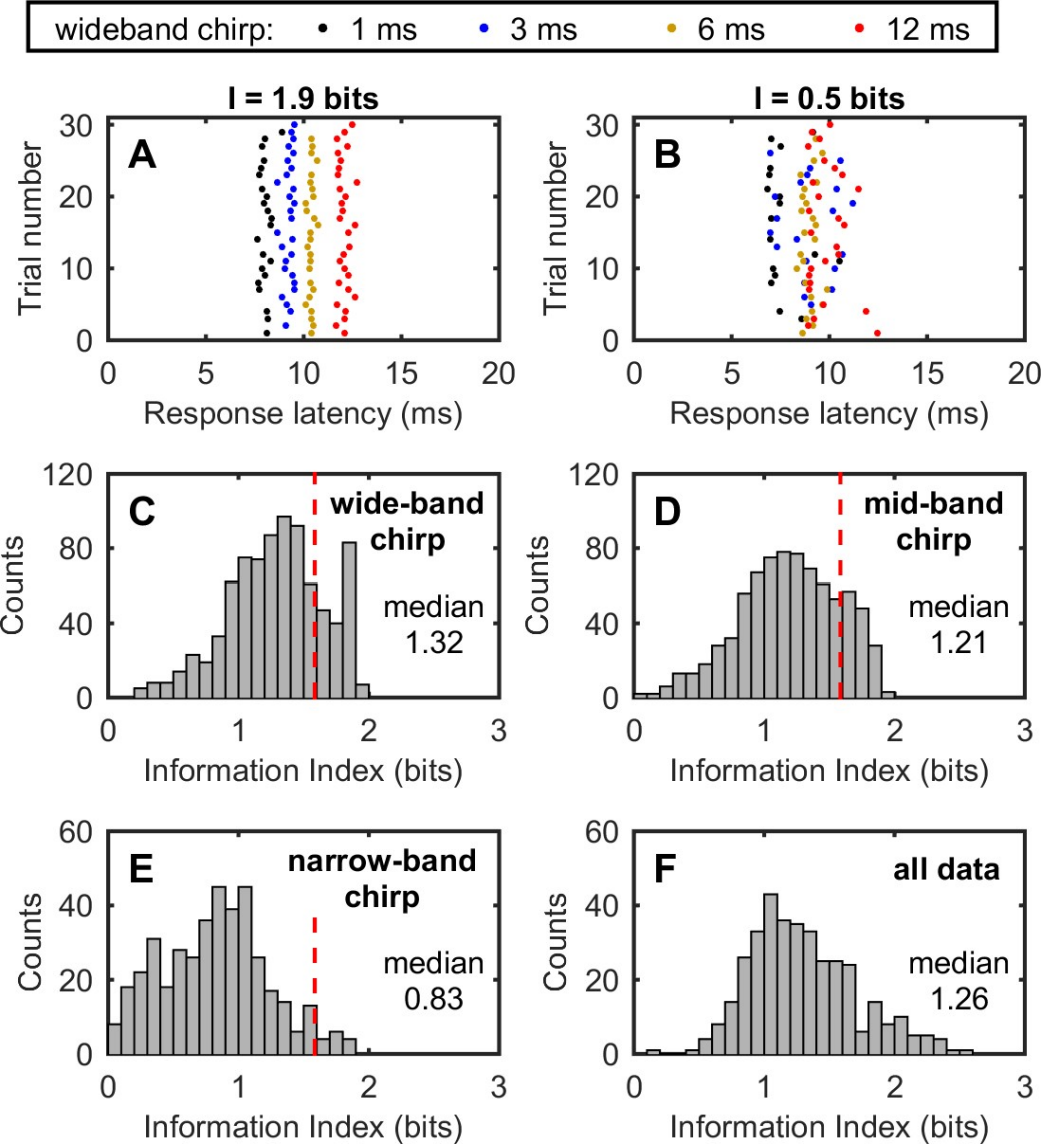
Information analysis of the timing of the EFP (200–600 Hz) evoked by simulated echolocation calls of different duration and bandwidth. (A, B) Two examples illustrating the mutual information calculated for wideband chirps (first harmonic, 25–55 kHz) of four different durations, i.e., 1 ms, 3 ms, 6 ms, and 12 ms. A mutual information of 1.9 bits in A shows that all four types of chirps can be distinguished nearly perfectly by the EFP response latency alone. By contrast, a mutual information of 0.5 bits in B shows that the four types of chirps cannot be categorized by the EFP response latency. Of note, a mutual information of 1 bit and 3 bits mean that two and eight types of signals can be distinguished, respectively. (C–F) Distribution of the mutual information calculated for the wideband chirps, midband chirps, narrowband chirps, and all chirp types, respectively. A median mutual information of 1.32 and 1.28 for the wideband chirps and the midband chirps indicates that the EFP response latency encodes substantial information about the duration of the chirps. Data for this figure are included in S1 Data. EFP, extracellular field potential.

The systematic change in EFP latency and precision with stimulus duration and bandwidth suggests that EFP latency itself could serve to code different stimulus parameters. To understand how much information is potentially encoded by the response latency of EFPs, we applied information theory and calculated the sample size–corrected Shannon mutual information [50]. First, we calculated the mutual information for each stimulus bandwidth, namely the wideband (25–55 kHz for the first harmonic), the midband (25–40 kHz for the first harmonic), and the narrowband (25–30 kHz for the first harmonic). Fig 5A shows an example with a relatively high mutual information, and Fig 5B shows an example with a relatively low mutual information. Of note, the theoretical maximum of the mutual information calculated for four stimulus types within each bandwidth category is 2 bits. Fig 5C–5F show the mutual information for all recording sites from all four bats. A maximum mutual information of 2.57 bits suggests that the response latency of EFPs from a single recording site can maximally distinguish six out of the 12 total stimulus types (Fig 5F). Nevertheless, within each bandwidth category, the response latency of EFPs from 185 (22.2%), 143 (18.2%), and 15 (3.6%) recording sites can distinguish at least three out of four stimulus types with a mutual information larger than 1.58 (red dashed line). Moreover, the wideband chirp featured the highest mutual information, followed by the midband chirp, and the narrowband chirp had the smallest mutual information (Wilcoxon rank sum test, all *P* < 0.001). These data suggested that the response latency of EFPs encodes more information about the duration of the stimulus type with a wider frequency bandwidth.

### Precise time difference estimation of simulated call–echo pairs by EFP

**Fig 6:**
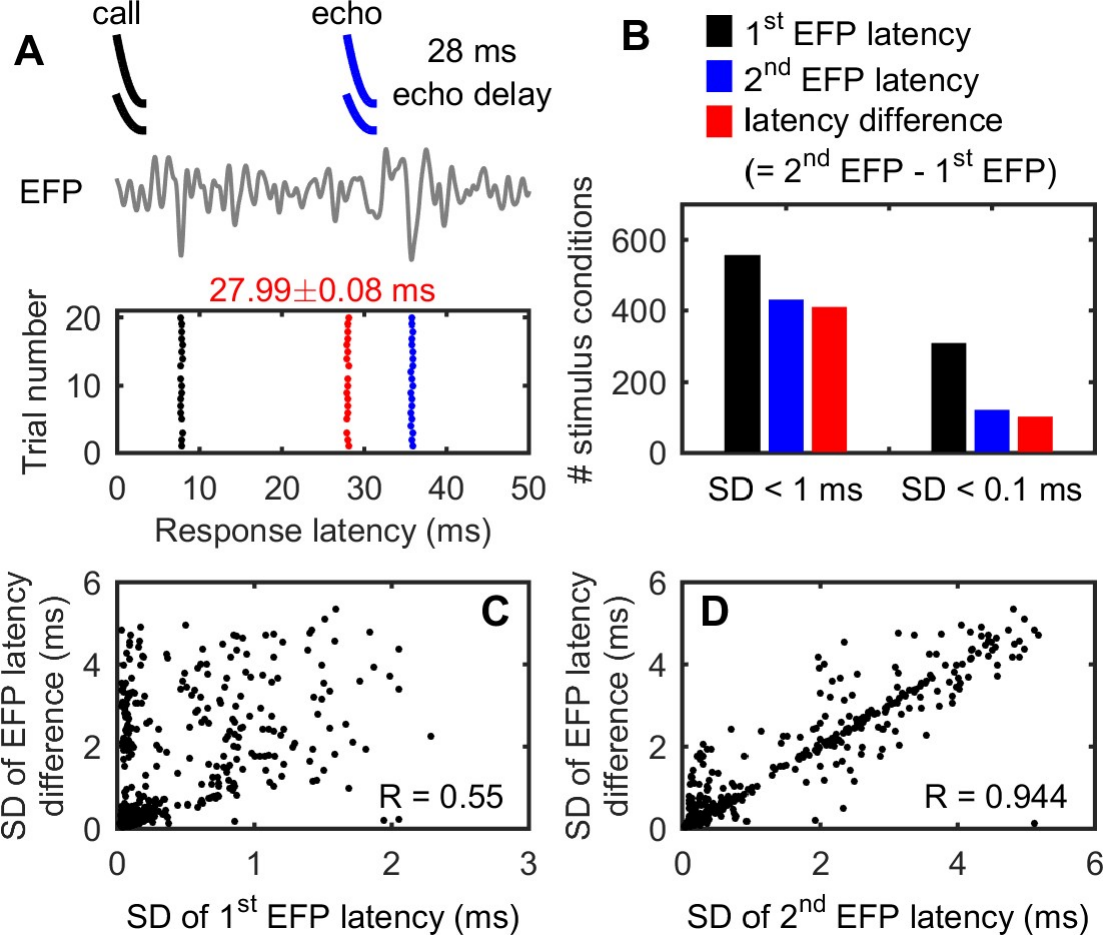
Precise time difference estimation of simulated call–echo pairs by EFP (200–600 Hz). (A) An example of the EFP waveform (gray in color) and raster display of the EFP latencies over 20 times of presentation of a simulated call–echo pair. The “call” was 3 ms in duration and was presented at an amplitude of 75-dB SPL. The “echo” was a 10-dB attenuated copy of the “call” and was presented 28 ms after the call. There were often two EFPs after each presentation (first responses, black dots; second responses, blue dots). The latency difference between the second response and the first response was calculated and displayed as red dots. (B) Relative abundance of the first EFP latency, second EFP latency, and the latency difference between these two. Data were based on a 90% detection reliability, and the total number of tested stimulus conditions was 684 from three bats. Thus, the latency difference estimated by the EFPs reached a submillisecond precision for 411 tested stimulus conditions (60%) and a precision higher than 100 μs for 103 tested stimulus conditions (15%). A stimulus condition refers to EFPs recorded at a particular recording depth for a particular stimulus type, e.g., a call–echo pair of 28-ms echo delay for a recording depth of 1,340 μm, as shown in panel A. The precision of the EFP latency difference was not determined by the precision of the first EFP latency (C) but by the precision of the second EFP latency (D). Data for this figure are included in S1 Data. EFP, extracellular field potential.

Echolocating bats use the time delay between an emitted call and returning echo to estimate the distance or range of objects in the environment [5,17,19]. For the big brown bat, the operating range of echolocation for prey detection is up to a few meters, which corresponds to echo delays of a few tens of milliseconds. To test whether the high-precision EFP evoked by single sounds can estimate the time difference of a pair of sounds that simulate the call–echo pairs used in echolocation tasks, we took neural recordings from the IC of three big brown bats (the same bats in Experiment 1) passively listening to simulated call–echo pairs. The calls were the “standard call” (Fig 6A) presented at an amplitude of 75-dB SPL. The echoes were attenuated versions of the standard call and were presented at either 25-, 45-, or 65-dB SPL and at delays between 2 and 30 ms following the call.

**Fig 7:**
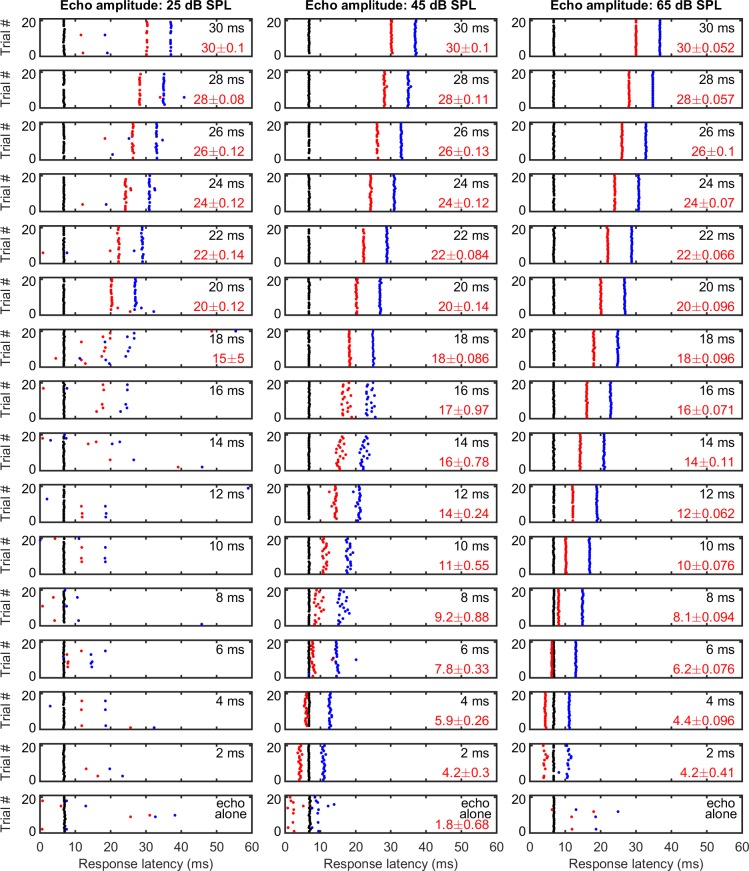
An example of time difference estimation for simulated call–echo pairs by EFP (200–600 Hz) over three echo amplitudes and a set of echo delays. The estimated time difference (red dots) increases with increasing echo delays and matches closely with the simulated echo delay at longer echo delays. For shorter echo delays, the estimated time differences were often larger. The “call” was 3 ms in duration and was presented at an amplitude of 75-dB SPL. The number in black at the top right corner of each panel indicates the actual echo delay of the simulated call–echo pair. The number in red (mean ± SD, only calculated for a sample size ≥10) shows the estimated time difference (red dots) by taking the difference between the second EFP latency (blue dots) and the first EFP latency (black dots). Each call–echo pair was presented 20 times. Data for this figure are included in S1 Data. EFP, extracellular field potential; SPL, sound pressure level.

Fig 6A shows an example of EFP responses to a call–echo pair over 20 presentations. The simulated echo delay was 28 ms. The echo was 10 dB weaker than the call, i.e., 65-dB SPL. After each presentation of the call–echo pair, there were often two EFPs. The first (black dots) and the second (blue dots) EFP responses occurred at a latency of 7.8 ± 0.075 ms and 35.8 ± 0.082 ms, respectively. The estimated echo delay (red dots) from the EFPs was 27.99 ± 0.08 ms, which is not only very precise across stimulus presentation but also aligns with the actual 28-ms echo delay. Fig 6B shows that across the 684 tested stimulus conditions from all three bats, the time difference estimated by EFPs showed a submillisecond precision in 411 (60%) stimulus conditions and showed a precision greater than 100 μs in 155 stimulus conditions (15%). Fig 6C and 6D shows that the precision of the stimulus time difference estimation by EFPs was largely constrained by the precision of the EFP response to the weaker echo (Fig 6D), not by the precision of the first EFP response to the more intense call (Fig 6C). In response to simulated call–echo pairs, the first EFP responses to the calls, with a median SD of 0.1 ms, were more precise in timing than the second EFP responses to the echoes, with a median SD of 0.29 ms (Wilcoxon signed-rank paired test, *P* < 0.001). Fig 7 shows the EFP responses at a single recording site to simulated call–echo pairs at three different echo amplitudes (25-, 45-, and 65-dB SPL) and at varying echo delays between 2 and 30 ms. These data show that at each echo amplitude, the estimated time difference of EFPs increases with the echo delay of the simulated call–echo pairs (from the bottom to the top) and achieves a precision greater than 100 μs for many stimulus conditions.

## Discussion

In this study, we have shown that the response latency of the EFP evoked by simulated echolocation calls shows high precision in the temporal registration of stimulus events, with latency variability as low as 17 μs. The EFPs, recorded in the IC of the awake, passively listening big brown bat, were generated from the synchronous firing of a population of neurons, and both the number of firing neurons and the tightness of neuronal synchrony influenced the EFP properties of response latency, precision (variability), and amplitude. We also provide evidence that the precision of the EFP depends on the spectrotemporal features of bat echolocation calls, and EFPs evoked by simulated call–echo pairs can precisely estimate echo delay.

There is a general consensus that spiking activity represents an important source of LFPs at frequencies higher than approximately 100 Hz, at least in the hippocampus [35,38,43,51,52]. Yet sources other than spiking activity, such as synaptic events, can also contribute to the LFP at higher frequencies [43,53]. Since the term LFP has been traditionally used to refer to EFPs at frequencies below 100–200 Hz, we adopted the term EFP [54] in this study to describe neural activity in a band between 200 and 600 Hz. Combining extracellular recording and mathematical modeling, we provide evidence that the EFPs in our IC recordings are generated by a population of neurons firing synchronously. Although the neurons that generate the EFP are likely located within the IC, one recent study raised the possibility that the EFP can be generated via axon bundles projecting from one brain region to another [54]. Specifically, the authors found that EFPs could be reliably recorded from axon bundles, and the properties of the EFPs were affected by the properties of the axons. This work implies that the source of EFPs recorded in the IC of bats in our study could be either from the local IC neurons, from axons passing through the IC, or a combination of the two.

One intriguing feature of the EFP is its remarkable precision in registering the timing of stimulus events. The most precise EFP evoked by the same acoustic stimulus over 30 trials showed an SD of 17 μs in response latency. Here, the reported 17-μs temporal precision might be an underestimation of the highest temporal precision achievable by EFPs in the IC of the big brown bat. Since the temporal precision of the EFP is determined by both neural synchrony and the number of firing neurons, based on Equation 2 in Fig 3C, one can predict a temporal precision of 3.2 μs from 1,000 neurons that each has 100-μs variability in response latency. Single neurons showing 100-μs temporal variability in response latency have been reported for the IC of the big brown bat [26]. Note that all the measured and predicted temporal precision reported above was for single EFP sites. Considering the possibility that the nervous system can combine information across multiple brain sites [28], submicrosecond temporal precision could be achieved, although the exact mechanisms of such population coding have yet to be identified.

Our study revealed two potential mechanisms that the nervous system can exploit to represent the timing of stimulus events with high precision: (1) many neurons responding to a given stimulus and (2) high synchrony of firing among these neurons. Increasing the tightness of neuronal synchrony is a general computational principle for modulating nervous system functions. At the single neuron level, precise spike timing can propagate or be maintained across synapses when multiple neurons fired in high synchrony [55–57]. At the synaptic level, highly synchronized presynaptic inputs are more effective at driving a neuron to fire [58] and generating spikes with higher temporal precision [59,60]. There is also evidence that neuronal synchrony is functionally linked to the behavioral performance of animals [61–63]. For example, Gutierrez and colleagues (2010) found that within the taste–reward circuit of rats, neurons that fired in synchrony with licking behavior exhibited greater cue discrimination than nonsynchronized neurons and that the magnitude of this effect increased with learning. On the other hand, the potential importance of a larger population of neurons for specific brain functions is a matter of debate [64]. Here, we identified one potential advantage of an increased neuronal population size in improving the temporal precision of the EFP. Nevertheless, it is worth noting that without neural synchrony, the population size of firing neurons itself contributes very little to improving the temporal precision of the EFP (Fig 3B, the low-synchrony scenario).

One crucial finding of this study is the functional relationship between the precision of registering the timing of stimulus events in the EFP and the spectrotemporal features of the echolocation calls of bats. During a foraging task, big brown bats use echolocations of long duration and narrowband frequency range to search for prey. A main function of the search-phase echolocation calls is prey detection, and long duration narrowband signals are well suited for this task [32,65]. Once a prey item is detected, insectivorous bats rapidly change the echolocation call structure by decreasing the call duration and increasing the frequency bandwidth while approaching the prey. One important function of the approach-phase echolocation calls is to track the position of the prey, which thus requires estimating target distance, i.e., measuring echo arrival time, with great precision. Moreover, there is extensive evidence showing that short broadband echolocation calls are well suited for precise measurements of echo arrival time [5,8,32,49]. Here, we found that the EFP evoked by echolocation calls of short duration and wideband spectrum showed the most precise registration of stimulus events, and by contrast, the EFP evoked by long-duration, narrowband echolocation calls showed the poorest temporal precision. Moreover, we have shown that the precise EFPs evoked by single sounds can also be used to estimate the time difference of simulated call–echo pairs with a precision as great as 45 μs, representing a feasible neural substrate for the 36–80-μs behavioral echo delay discrimination by echolocating bats [4]. The precision of time difference estimation of the simulated call–echo pairs was largely constrained by the second EFP responses to the weaker echoes, which were less precise than the first EFP responses to the more intense calls. This finding suggests that echo amplitude might play a role in influencing the precision of target ranging and points to the potential importance of stabilizing echo amplitude for target ranging. Interestingly, there is an accumulating body of evidence that echolocating bats maintain a relatively constant echo amplitude while approaching a target [66,4,67,68]. Thus, the temporal precision of the EFP, as a marker for acoustic events, provides a window to neural computational principles that might underlie the accurate echo delay discrimination behavior of echolocation tasks.

The findings reported here raise several important questions that can be answered by further research. One critical question concerns the mechanisms by which the brain makes use of the neural events underlying the precise EFPs. At this time, knowledge is lacking about the contribution of the biophysical properties of single neurons and/or biochemical environments to the EFP. Because EFPs are generated by synchronous firing in a population of neurons, the high-precision EFP may be a proxy for population coding across single neurons. Thus, the question of how the brain might extract and use information carried by EFPs could be approached through population analysis of stimulus-evoked activity in pools of single neurons. We also conjecture that inhibition may play an important role in generating the precise EFPs, which is known to underlie precise interaural time difference estimation in mammals [69]. Specifically, many properties of IC neurons, such as selectivity for interaural intensity difference, sound frequency, and sound duration, are generated by convergent inhibitory and excitatory inputs, as revealed by the intracellular recording of postsynaptic potentials [70–72]. Both intracellular recording and application of inhibitory neurotransmitter antagonists in the IC could provide a first step to investigate the role of inhibition in shaping EFP properties.

Going beyond the implications for understanding mechanisms of precise target ranging performance in echolocating bats, the high-precision EFP bears general relevance for understanding stimulus coding in the brain across species. Precise timing of neural signals could encode rich information that is relevant to a variety of brain functions [73]. For example, the temporal precision of neural events may be a key property in categorizing sound features by the auditory system [74,75]. Indeed, the information theoretic analysis showed that EFPs from many recording sites in the bat IC could unambiguously differentiate three out of four simulated echolocation calls of different durations. By extension, EFP analysis could generate insights into mechanisms supporting other auditory behaviors, such as acoustic communication, scene analysis, and spectrotemporal discrimination [76]. Although specialized neural mechanisms for temporal processing have been revealed in species that use stimulus timing in natural behaviors, such as barn owls [9] and electric fish [11], the extent to which diverse species share common mechanisms to achieve high temporal precision is an important open question. We propose that the computational principle of synchronized firing across a population of neurons represents a general mechanism for the nervous system to register precisely the timing of sensory events.

## Materials and methods

### Ethics statement

Big brown bats, *Eptesicus fuscus*, collected in the state of Maryland under a permit issued by the Department of Natural Resources were used as subjects (permit number 55440). The Johns Hopkins University’s Institutional Animal Care and Use Committee approved all the procedures used for this study (protocol number BA14A111). The protocols are in compliance with the Animal Welfare Act regulations and Public Health Service Policy. The university maintains accreditation by the Association for the Assessment and Accreditation of Laboratory Animal Care International.

### Neural recording

Extracellular recordings were made from auditory neurons in the IC of seven adult big brown bats (one male, six females) in an acoustic booth. In the big brown bat, the IC sits on the dorsal surface of the brain and is up to approximately 2 mm in length both anterior-posteriorly and dorsoventrally. On the day of neural recording, the bat was placed into a custom-made bat holder, its head was immobilized via a headpost, and a craniotomy of <1-mm^2^ size above the central IC was made with a scalpel under a microscope. Multiple penetrations were made to the IC of the same bat in different recording days and bats were recorded between a few days and more than a month. Within a single penetration, the probe was systematically advanced dorsoventrally from the surface down to approximately 1,800 μm using a hydraulic Microdrive (FHC). Extracellular potentials were recorded by a silicon probe from Neuronexus that had the 1 × 16 arrangement of recording sites, with an intersite separation of 50 μm. The probe thickness was 25 μm and the site area of the probe was either 177 μm^2^ or 703 μm^2^. On the day of the headpost surgery, under isoflurane inhalation anesthesia, part of the skin and the temporal muscles overlying the IC were removed, and a custom headpost was attached to the bone at the midline using cyanoacrylate gel.

During neural recording, the awake bat was passively listening to the broadcasts of simulated echolocation calls or call–echo pairs from a custom electrostatic loudspeaker (1-cm diaphragm) placed 60 cm away from the contralateral ear of the recording IC at an angle of approximately 30° from the middle line. Digital echolocation calls were generated from customized LabVIEW scripts and played at a sampling rate of 1 MHz using the data acquisition card from National Instrument (PXIe 6358). We achieved a flat frequency response of the playback system for the frequency range of 20–100 kHz (±1 dB) by digitally compensating for the uneven frequency response with its compensatory impulse response [77,78]. The compensatory impulse response was computed using the Maximum Length Sequence method based on 5 seconds white noise recordings with a one-fourth–inch measurement microphone (Model 7016, ACO, United States). Acoustic stimuli were played at an amplitude of 70-dB SPL (root mean square) unless otherwise stated. Each acoustic stimulus was repeated at least 20 times. Specifically, the standard 3-ms echolocation call (Fig 1A, first harmonic sweeps down from 55 kHz to 25 kHz, and the second harmonic from 110 kHz to 50 kHz) was repeated 80 times in the first experiment, and all other stimuli, such as white noise or time-inversed echolocation calls shown in Fig 1D, were repeated 20 times. In the second experiment that tested the functional significance of the EFPs, each stimulus was repeated 30 times. The call–echo pairs in the third experiment were repeated 20 times. The time interval between stimulus presentations was 300 ms, and the order of stimulus presentation was randomized for each experiment. The neural recording was digitized at a sampling rate of 40 kHz using a Plexon system of 64 analog channels. The original neural signal was amplified by a factor of 20 times before digitization but later was restored to the correct scale during data analysis. Neural recording and sound broadcasting were synchronized via a transistor–transistor logic (TTL) signal outputted from a second analog output channel of the National Instrument card each time when an acoustic stimulus was broadcast, and the TTL signal was directly recorded by an analog input channel of the Plexon system. More details on the animal surgical preparation and neural recording can be found in Macias and colleagues (2018) [79].

### Data analysis

All neural recordings were batch processed in Matlab (R2015a, Mathworks). The general steps of data processing include (1) band-pass filtering the original recording with Elliptic filters from the Wave_clus algorithms [42]; (2) an adaptive threshold, six times of the background noise level, based on the same equation from Quiroga and colleagues (2004), was used to identify spikes and the peaks of EFPs; and (3) the onset time of the acoustic stimuli was identified based on the TTL signal, then the response latency of the MUA or the EFP was computed from the first negative peak. Negative spikes were the dominant observations across our recording. We quantified the precision or variability of the peaks of the EFPs with the SD. Data points were considered as outliers and excluded from analysis if their values were greater than *q*_3_ + *w* × (*q*_3_ − *q*_1_) or less than *q*_1_ − *w* × (*q*_3_ − *q*_1_), where *w* was set to 1.2 and *q*_1_ and *q*_3_ were the 25th and 75th percentiles of the sample data, respectively. We applied the outlier exclusion criterion, as we are particularly interested in how precise in timing the EFPs can potentially achieve. Moreover, outliers can greatly bias the estimations of the mean and the SD, which are the principal measures in the current study. Although outlier exclusion results in less variation in the measured EFP latencies and the corresponding SD of the EFPs, it is important to note that the reported EFPs were based on a 90% detection reliability criterion (see Results above), which limited the outliers in <10% of the data points. Note that although a sampling rate of 40 kHz results in a temporal resolution of 25 μs, the SD of a 25-μs resolution sampling system can be as small as approximately 5 μs [80]. Thus, the reported best temporal precision of 17 μs is well within the resolution of our data sampling system.

### Biophysical simulation

A cell model, L4_SS_cADpyr230_1, was downloaded from the Human Brain Project's Neocortical Microcircuitry (https://bbp.epfl.ch/nmc-portal/welcome) [44]. We constructed cell populations consisting of 10–1,000 cells, by randomly rotating and distributing instances of the same cell model with a uniform cell density of 200,000 neurons per mm^3^, within a disc of 100-μm thickness. Because of the fixed cell density, the radius of the disc was dependent on the population size.

Each cell in the population received 100 conductance-based excitatory synaptic inputs randomly distributed across the cell membrane with a uniform area weighted density. The synapses were implemented using a double exponential function (Exp2Syn in NEURON) with the rise and decay time constants of 1 ms and 3 ms, respectively, and a reversal potential of 0 mV. The synaptic weights were normally distributed around 0.2 nS with a standard deviation of 0.04 nS. The arrival of the 100 synaptic inputs was normally distributed with a mean value of T = 230 ms after stimulation onset, with a standard deviation of 0.25 ms. This evoked an action potential in the cell model, and after the extracellular action potential was calculated, a time window of ±15 ms around the maximum value of the somatic membrane potential was extracted from the extracellular action potential and saved to file. The time resolution of the simulation was 1/32 ms.

Extracellular potentials were calculated using LFPy2.0 (http://EFPy.github.io/) [81], which runs on the NEURON simulator [82]. For the calculation, each cell compartment was treated as a line source, except for the somatic compartments, which were treated as a point source. The extracellular conductivity was set to 0.3 S/m [83]. The extracellular potential was calculated in the center of the cell population.

Each of the calculated extracellular action potentials was randomly jittered in time, with different SDs for the jitter, and summed to produce the population signal. For each of the different tested SDs for the jitter, 100 trials of the population signal were calculated. The signals were filtered with elliptic filters (scipy.signal.ellip) to obtain the EFP and MUA. The first peak of the filtered signal was identified as the first negative crossing below a threshold of six times the SD of the signal.

All code used for this project is available from https://github.com/torbjone/sharp_wave_ripples/.

Details of the mathematical model shown in Fig 3, spike sorting, and the information analysis were presented in the S1 Text.

## Supporting information

S1 Text Details for the mathematical model, spike sorting, and the information analysis. (DOCX)

S1 Data Excel spreadsheet containing, in separate sheets, the underlying numerical data for Figs 2A–2G, 4B, 4C, 5A–5F, 6A–6D, 7, S1, S2A and S2B. (XLSX)

S1 Fig EFPs in the IC of the big brown bat are selective to the spectrotemporal features of acoustic stimuli. Data are based on 202 recording sites from three bats, in which at least one of the six types of the acoustic stimuli evoked ≥5 EFP responses over the 20 presentations, i.e., 25% response probability. The recording sites were organized dorsoventrally between a recording depth of 30 and 1,800 μm. Data for this figure is included in S1 Data. EFP, extracellular field potential; IC, inferior colliculus.
(TIF)

S2 Fig Functional correlate between EFPs and bat sonar behavior. (A, B) Echolocation calls of short duration and wide frequency range evoke EFPs of highest temporal precision and shortest response latency. For each condition, the data were from the EFP sites whose temporal precision were among the top 50% and were reliably detected for at least 90% of the trials. The number above each boxplot is the median and the number below the boxplot in A is the number of recording sites (i.e., sample size). Different from Fig 4, data from the same recording sites are presented for different stimulus durations, as can be seen from the same sample size. A sample size of three shows that it was very rare to find recording sites of reliable EFPs that were evoked by narrowband echolocation calls of multiple durations. Statistical significance levels of the nonparametric rank sum test between the data group and the neighboring left data group are indicated by asterisks (*P* < 0.05*; *P* < 0.01**; *P* < 0.001***) or “ns” (*P* > 0.05). Data for this figure is included in S1 Data. EFP, extracellular field potential.
(TIF)

S1 Movie Echolocation calls evoke precise EFPs. Top panel to the left shows the spectrogram of the echolocation call. Middle panel to the left shows the EFP (20–600 Hz) of the neural recording. Bottom panel to the left shows the spikes (600–3,000 Hz). The panel to the right is a raster plot that displays the response latency of the EFP over 20 trials. Neural recordings from 20 trials were combined and for each trial, the data included neural recordings 50 ms before and 50 ms after the stimulus. Both the audio of the echolocation calls and the neural recordings were slowed down by a factor of 20 times. EFP, extracellular field potential.
(MP4)

## Abbreviations

EFP: extracellular field potentials
FM: frequency modulated
IC: inferior colliculus
LFP: local field potential
MUA: multiunit activity
SD: standard deviation
SNR: signal-to-noise ratio
SPL: sound pressure level
TTL: transistor–transistor logic
