# Supplementary material for: Neural timing of stimulus events with microsecond precision
Source: PLoS Biol. 2018 Oct 26;16(10):e2006422. doi: 10.1371/journal.pbio.2006422 (PMC6221347; doi:10.1371/journal.pbio.2006422)
Supplement: S1 Text — (DOCX) [file pbio.2006422.s001.docx]

**S1 Text**

**Mathematical Model**

Here we provide an approximate formula for temporal population coding that explains the properties of the EFPs.

Let function describe the standard shape of a spike waveform and assume that the extracellular field potential $L(t)$ is a sum of $n$ copies of the same spike waveform with random time jitters. More precisely, we assume that

where are the time jitters of spike 1 to spike $n$. Note that if the peak position of the spike is at the time $t_{0}$, then the peak position of is $t_{0}+\varepsilon_{i}$. Here we only consider the relative timing of the extracellular field potential $L(t)$ relative to the standard spike waveform , and there is no need to specify the exact value of $t_{0}$.

*First-order approximation.* There is a useful approximation formula in the limit of small time-jitters. As a first-order approximation, the location field potential can be written as

where $\bar{\varepsilon}=\frac{1}{n}\sum_{i=1}^{n} \varepsilon_{i}$

is the average of the jitters. In other words, the extracellular field potential waveform is approximately an amplified and time-shifted version of the standard spike waveform. The amplification factor is $n$, which means that the amplitude of the extracellular field potential is approximately $n$ times the standard spike waveform. Furthermore, the time shift is$\bar{\varepsilon}$, which is the overall equivalent time jitter of the extracellular field potential.

*Proof*: By the first-order Taylor expansion we have . This is a good approximation when the jitter $\varepsilon_{i}$ is very small (compared with the width of the spike waveform). Thus

where the approximation in the last step also follows from Taylor expansion.

Two useful results can be derived immediately.

First, the peak position of the extracellular field potential $L(t)$ is approximately given by $t_{0}+\bar{\varepsilon}$ where $t_{0}$ is the peak position of the spike . In other words, the jitter of the extracellular field potential peak is approximately equal to $\bar{\varepsilon}$.

Second, the standard deviation of the jitter of the peak position of the location field potential is given by

$$\sqrt{\left\langle\bar{\varepsilon}^{2}\right\rangle}=\sigma/\sqrt{n}\text{ [2]}$$

where $\sigma$ is the standard deviation of the time jitter of individual spikes and $n$ is the total number of spikes. In other words, the peak of the extracellular field potential has less jitter than the individual spikes, and the jitter reduces as the number of spike increases.

To see this, we assume that the jitters of the spikes are independent and identically distributed random variables with zero means. That is . We also assume that they have the same variance. It follows from these assumptions and the relation $\bar{\varepsilon}=\frac{1}{n}\sum_{i=1}^{n} \varepsilon_{i}$ that $\left\langle\bar{\varepsilon}\right\rangle=0$ and $\left\langle\bar{\varepsilon}^{2}\right\rangle=\sigma^{2}/n$.

*Second-order approximation.* A second-order formula based on Taylor expansion can be derived as follows:

where $\bar{\varepsilon}=\frac{1}{n}\sum_{i=1}^{n} \varepsilon_{i}$ is the sample mean and $\bar{\sigma}^{2}=\frac{1}{n}\sum_{i=1}^{n} \varepsilon_{i}^{2}-\bar{\varepsilon}^{2}$ is the sample variance of the jitters of the spike waveforms. The second-order formula should be more accurate than the first-order formula. But, unlike the first-order formula, the amplitude and the jitter of the final extracellular field potential are implicit in the formula and need to be solved numerically.

*Comparison with numerical simulations*. The first-order approximation formulas [1] and [2] as shown in **Fig. 3C** are most accurate when the jitters are much smaller than the spike width. Based on our second-order approximation theory presented above, we expect that an increase of the jitters should lead to a *reduction* of the EFP amplitude, whose approximate linear relationship with *n*, the number of firing neurons, should still be maintained. This is because in formula [3], the second derivative is expected to be negative at the peak, which implies a reduction of the amplitude of $L(t)$, and the factor $\bar{\sigma}^{2}$ implies that the reduction becomes more pronounced as the jitter variance increases. As shown in **Fig. 2B**, formulas [1] and [2] provides a reasonable first approximation to our numerical simulation results. To accommodate possible second-order effects, the theoretical curves in **Fig. 2B** were based on a single-parameter fit to the simulation data. For the amplitude, the coefficient obtained from the fitting was about 37% smaller than that expected from a single spike. Similarly, for the SD of latency, the reduction of the coefficient was about 34%.

*Synaptic potentials (downstream readout).* Similarly, the total postsynaptic potential evoked by $n$ spikes with time jitters $\varepsilon_{i}$ can be written as

$$v(t)=\sum_{i=1}^{n} \text{epsp}(t-\varepsilon_{i})$$

where epsp(*t*) is the EPSP evoked by a single spike. The approximate formulas of time population coding considered above can be applied to this equation as well.

**Spike Sorting**

To gain some understanding of the temporal precision of IC neurons in this study, we sorted MUA of high SNR into putative single units. Because our main goal was for high-quality LFPs, which are generated by multiple neurons firing in synchrony, sorting these temporally overlapping spikes represented a big challenge. To improve the quality of spike sorting, multiple quality control measures were implemented. First, we used the Wave_Clus software [1] to manually sort the MUA of high SNR into single units, with the spike detection threshold set as 8 times of the background noise level, followed by visual inspection of the spike shapes of the sorted units. Subsequently, the sorted unit of the greatest spike counts was regarded as putative single units, from which we calculated the SD of the 1^st^ spike latency in response to the standard chirp of 3 ms duration. Moreover, only the neurons that showed > 10% of the responses to the standard chirp were included for the SD analysis, which resulted in a minimum sample size of 8. Lastly, we used the < 3 ms inter-spike interval as an additional index to control the quality of the spike sorting, as a high percentage of < 3 ms inter-spike intervals indicate low-quality of spike sorting [1]. Specifically, all the single units reported in this study contained < 5% of < 3 ms inter-spike-intervals. In total, 59 single units from the 1^st^ Experiment were identified. 20 single units were identified when a 1% threshold of < 3 ms inter-spike interval was chosen.

**Information Analysis**

We applied information theory to quantify the amount of information potentially encoded by the EFP response latency about the stimulus types using the data collected in **Experiment 2**. In this experiment, we recorded EFPs with simulated echolocation calls of three different bandwidths (wide-band, mid-band, and narrow-band) and four different durations (1 ms, 3 ms, 6 ms, and 12 ms), with a total of 12 stimulus types. We calculated Shannon mutual information between stimulus and response as follows:

$$I=\sum_{i=1}^{S} \sum_{j=1}^{R} p(s_{i},r_{j})\log_{2}\frac{p(s_{i},r_{j})}{p(s_{i})p(r_{j})}-\frac{(S-1)(R-1)}{2N\ln2}$$

where $p(s_{i},r_{j})$ is the joint probability density for stimulus $s_{i}$ and response $r_{j}$, $p(s_{i})=\sum_{j=1}^{R} p(s_{i},r_{j})$ and $p(r_{j})=\sum_{i=1}^{S} p(s_{i},r_{j})$ are the marginal distributions, the last term is a correction term for reducing the upward bias caused by finite sample size [2], $S$ is the number of bins for stimulus (total number of stimulus types), $R$ is the number of bins for response latency, and $N$ is the total number of trials (stimulus types $\times$ number of repeats). We analyzed the mutual information for each recording site and each bandwidth type (i.e. 4 total stimulus types) as well as for all stimulus types (i.e. 12 total stimulus types). The total number of the bins for response latency was determined by both the range of the EFP response latency (i.e. the min and the max of the response latency) and the temporal resolution (bin width). For each recording site, we calculated the mutual information for 20 temporal resolutions from 0.1 to 2 ms at a step of 0.1 ms. Although mutual information did not depend sensitively on the temporal resolution especially for intermediate values, we observed that the exact value of mutual information without correction tended to slightly decrease while the sample-size corrected mutual information tended to increase, as the temporal bin width increases and the total number of bins decreases. The mutual information value reported in this study was the corrected mutual information from the temporal resolution at which the mean value of the original mutual information and the corrected mutual information was the largest.

**Reference**

1. Quiroga RQ, Nadasdy Z, Ben-Shaul Y. Unsupervised spike detection and sorting with wavelets and superparamagnetic clustering. Neural Comput. 2004;16: 1661–1687. doi:10.1162/089976604774201631

2. Treves A, Panzeri S. The upward bias in measures of information derived from limited data samples. Neural Comput. 1995;7: 399–407. doi:10.1162/neco.1995.7.2.399
