# Supplementary figures and images for: Neural timing of stimulus events with microsecond precision
Source: PLoS Biol. 2018 Oct 26;16(10):e2006422. doi: 10.1371/journal.pbio.2006422 (PMC6221347; doi:10.1371/journal.pbio.2006422)

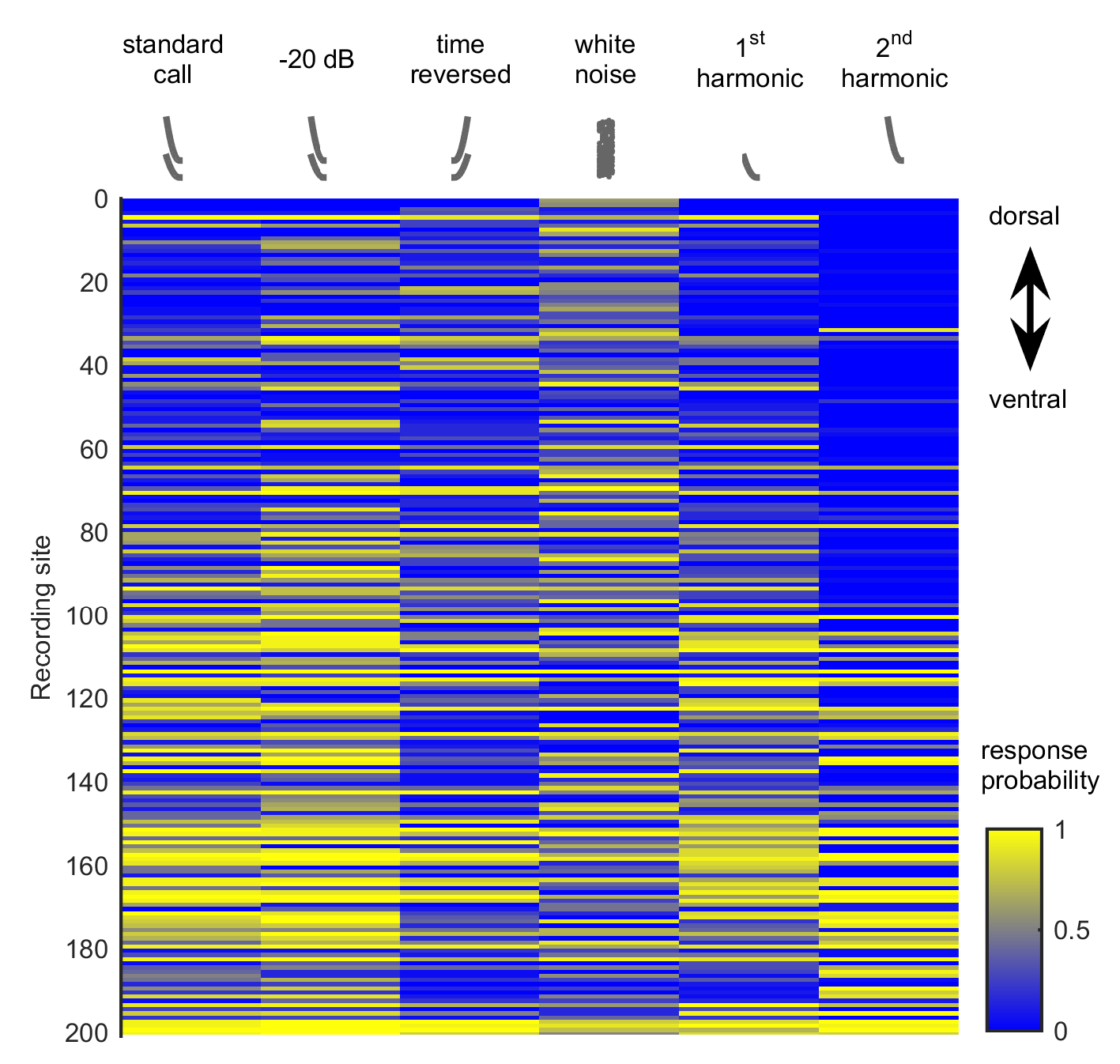

Supplement: S1 Fig — Data are based on 202 recording sites from three bats, in which at least one of the six types of the acoustic stimuli evoked ≥5 EFP responses over the 20 presentations, i.e., 25% response probability. The recording sites were organized dorsoventrally between a recording depth of 30 and 1,800 μm. Data for this figure is included in S1 Data. EFP, extracellular field potential; IC, inferior colliculus. (TIF) [file pbio.2006422.s003.tif]

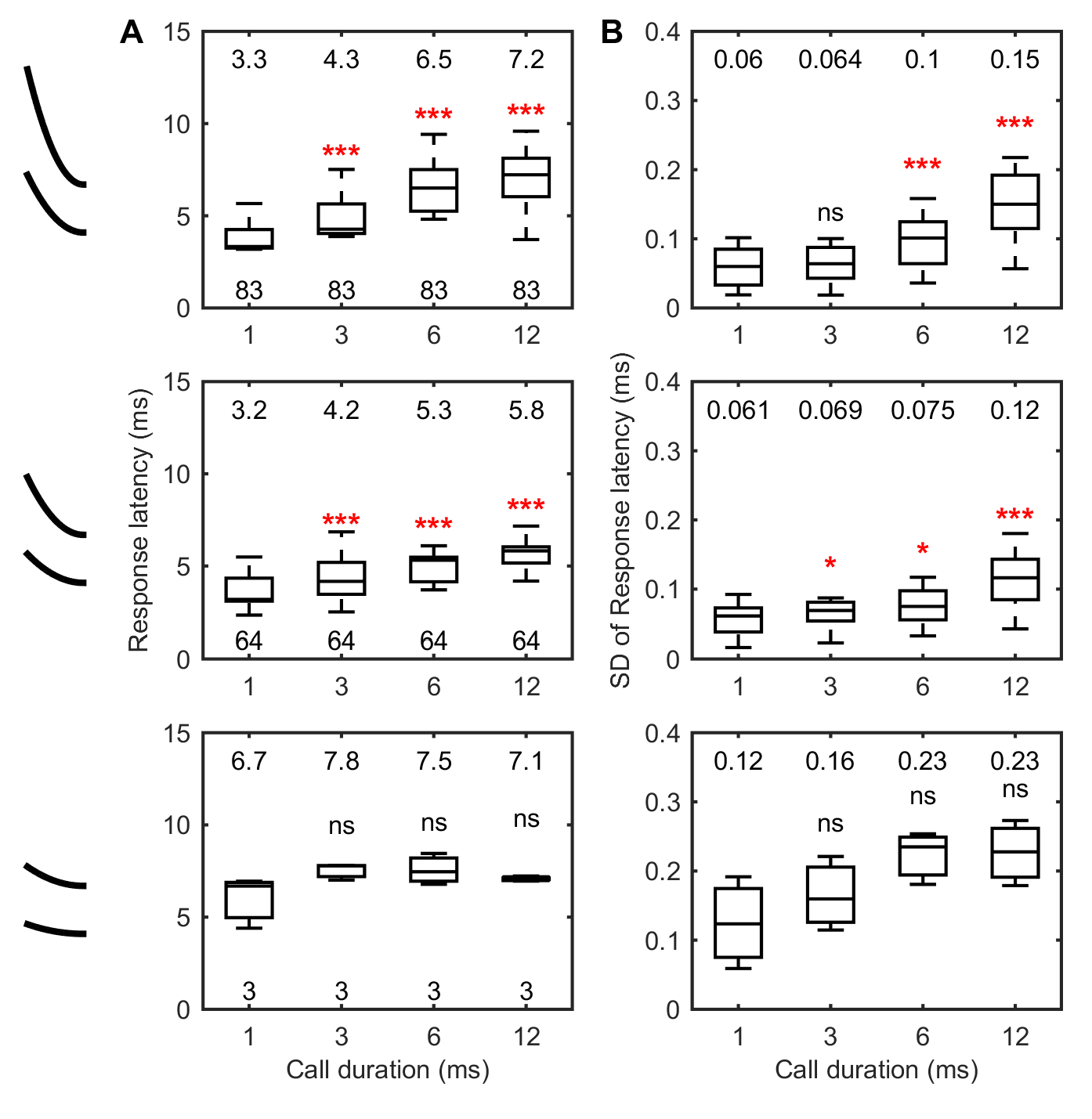

Supplement: S2 Fig — (A, B) Echolocation calls of short duration and wide frequency range evoke EFPs of highest temporal precision and shortest response latency. For each condition, the data were from the EFP sites whose temporal precision were among the top 50% and were reliably detected for at least 90% of the trials. The number above each boxplot is the median and the number below the boxplot in A is the number of recording sites (i.e., sample size). Different from Fig 4, data from the same recording sites are presented for different stimulus durations, as can be seen from the same sample size. A sample size of three shows that it was very rare to find recording sites of reliable EFPs that were evoked by narrowband echolocation calls of multiple durations. Statistical significance levels of the nonparametric rank sum test between the data group and the neighboring left data group are indicated by asterisks (P < 0.05*; P < 0.01**; P < 0.001***) or “ns” (P > 0.05). Data for this figure is included in S1 Data. EFP, extracellular field potential. (TIF) [file pbio.2006422.s004.tif]
